# Ultra-sensitive protein detection via Single Molecule Arrays towards early stage cancer monitoring

**DOI:** 10.1038/srep11034

**Published:** 2015-06-08

**Authors:** Stephanie M. Schubert, Lisa M. Arendt, Wenhui Zhou, Shazia Baig, Stephanie R. Walter, Rachel J. Buchsbaum, Charlotte Kuperwasser, David R. Walt

**Affiliations:** 1 Department of Chemistry, Tufts University, Medford, MA 02155; 2 Department of Developmental, Molecular, and Chemical Biology, Tufts University School of Medicine, Boston, MA 02111; 3 Department of Medicine, Tufts Medical Center, Boston, MA 02111; 4 Molecular Oncology Research Institute, Tufts Medical Center, Boston, MA 02111

## Abstract

The early diagnosis of cancers and continued monitoring of tumor growth would be greatly facilitated by the development of a blood-based, non-invasive, screening technique for early cancer detection. Current technologies for cancer screening and detection typically rely on imaging techniques or blood tests that are not accurate or sensitive enough to definitively diagnose cancer at its earliest stages or predict biologic outcomes. By utilizing Single Molecule Arrays (SiMoA), an ultra-sensitive enzyme-linked immunosorbent assay (ELISA) technique, we were able to measure increasing levels of prostate specific antigen (PSA) within murine serum over time, which we attribute to tumor development. The measured concentrations of PSA were well below the detectable limits of both a leading clinical diagnostic PSA ELISA assay as well as a commercial ultra-sensitive PSA assay. Our work benchmarks the role of SiMoA as a vital tool in monitoring previously non-detectable protein biomarkers in serum for early cancer detection and offers significant potential as a non-invasive platform for the monitoring of early stage cancer.

The ultimate goal in cancer diagnostics is to develop tools to detect harmful disease states as early as possible. The ability to detect and treat cancer at early stages is of paramount importance in clinical practice, because for multiple types of cancer, including prostate^1^, breast^2^, ovarian^3^, and colon^4^, it has been demonstrated that early detection leads to both improved prognosis and improved survival rates. Tumor biomarkers typically consist of protein, RNA, or DNA abnormalities produced by either cancer cells or by the body in response to the presence of cancer^5^. While cancer-screening methods continue to push the boundaries in terms of detection limits, the development of ultrasensitive blood-based detection methods for cancer and cancer biomarkers could significantly benefit cancer diagnostics by enabling earlier detection than is currently possible. Furthermore, the use of biomarkers in blood-based diagnostics has the potential for differentiating biologically relevant disease from tumors that may never become symptomatic—a dimension that image-based diagnostics lack^6^. Unlike methods that require tumor tissue, such as tissue genotyping^7^, measuring protein biomarkers in blood represents a less invasive process.

ELISAs are the most common test available for measuring protein concentrations in blood; however, due to a lack of sensitivity, ELISAs may not be able to detect clinically relevant protein biomarkers in serum at very low levels^8^. In order to detect ultra-low concentrations of the cancer biomarker PSA, we utilized SiMoA, a recently developed ultra-sensitive ELISA based on single molecule counting technology^9^. The fundamental theory of SiMoA has been previously published by Chang and coworkers^10^. The limit of detection (LOD) of a leading clinical diagnostic PSA ELISA assay (Siemens, usa.healthcare.siemens.com) is 100 pg/mL and the LOD of commercially available ultra-sensitive ELISA-based PSA tests is between 3–10 pg/mL^11^. The experiments described in this paper are compared to these two benchmarks. We obtained a LOD of 0.005 pg/mL for PSA using SiMoA, which is significantly more sensitive than ELISA and is comparable to previous SiMoA work^9^. Other literature reports have also demonstrated sensitive PSA tests, including recent work by Liu *et al.* where they utilized gold nanoparticles to create a colorimetric ultra-sensitive assay for PSA with a LOD of 0.0031 pg/mL^12^.

Recently, several groups have produced ultra-sensitive assays for the detection of various protein biomarkers. Notable works have utilized electrochemical microfluidic arrays^13^, electrochemical immunosensors with gold nanoparticles functionalized with magnetic multi-walled carbon nanotubes^14^, and novel laser-induced fluorescence systems^15^ to detect cancer biomarkers. Although these reports advanced the field of ultra-sensitive biomarker detection, many of them require complicated assay set-ups, lengthy preparation, or are potentially subject to sensor fouling.

SiMoA has been previously implemented in studies for monitoring recurrence of prostate cancer after radical prostatectomy^16^, as well as tumor necrosis factor-alpha (TNF-α) and interleukin 6 (IL-6) for monitoring therapeutic efficacy in Crohn’s disease^17^. Importantly, recent work by Warren *et al.* describes the use of SiMoA to noninvasively discriminate between mice with and without thrombosis by detecting microdosed disease-tailored nanoparticles at ultra-low levels^18^. SiMoA represents an already commercialized method that is simple, straightforward, and can be performed in a fully automated instrument in a few hours with minimal preparation. These attributes make SiMoA an ideal tool for implementation in clinical settings.

We chose to use PSA as a cancer biomarker for a proof-of-concept study for early cancer detection due to the extremely sensitive SiMoA LOD and its high reproducibility. This work describes the use of a prostate cancer cell line to create a novel murine xenograft model to monitor tumor formation in mice. Since the only source of human PSA within the mouse model are the human cells that are injected and then replicated in the mouse, the concentration of PSA in the mouse’s bloodstream should correspond to the number of tumor cells that may ultimately form tumors. We demonstrate that due to its high sensitivity, SiMoA can be used to detect the presence of nascent tumors at a much earlier stage than is possible with any other protein assay. Although this work is demonstrated with PSA, it should be applicable to any biomarker associated with tumor growth that is found in the blood.

## Results

### Mouse Model Design

Our goal was to create a mouse model for prostate cancer and to track the progression of serum PSA concentration using SiMoA technology prior to the formation of palpable tumors. Cell line-based mouse xenografts are commonly used in cancer research and are employed in this study using LNCaP cells. LNCaP cells are an epithelial cell line that originated from a metastatic lesion of a human prostatic adenocarcinoma and are commonly used in prostate cancer research. The doubling time for this cell line *in vitro* is approximately 60 hours^19^.

Previous studies have demonstrated that upon inoculation of three million LNCaP cells, tumors will form in male immunocompromised mice within eight weeks^19^. Building on this work, initial experiments were performed to determine a baseline level of PSA in the serum of mice inoculated with typical concentrations of LNCaP cells. In the first study, two mice were inoculated with 4 × 10^6^ LNCaP cells and terminal bleeds were collected after four weeks. At end stage, tumors from these mice measured about 10 mm in diameter (data not shown). Serum PSA concentrations were extremely high, measuring approximately 30,000 and 78,000 pg/mL for each mouse (Supplementary Fig. 1). These values are significantly higher than the LOD of SiMoA and of traditional ELISA PSA assays.

Since inoculating mice with 4 × 10^6^ LNCaP cells led to serum PSA concentrations that were well above the range of SiMoA, the number of cells used for inoculation was reduced. Consequently, 19 male NOD/SCID mice were inoculated with 1 × 10^6^ LNCaP cells subcutaneously to test how early SiMoA could detect PSA within the serum. Terminal bleeds were taken daily for 19 days, beginning the day after inoculation. It is important to note that only a single measurement could be obtained from each mouse due to the volume of blood required to perform the analysis; therefore, the data presented here are from three different mice at each time point. Although several measurements were below the detection limit, PSA was detected in the serum of one mouse after only three days (see Supplementary Fig. 2). A general trend of increasing PSA concentration over the 19 days was observed and all values were below the detection limit of standard ELISA. Ultra-sensitive ELISA was able to detect PSA in samples from days 12 and 16-19. None of the mice in this study developed tumors; however, the study only spanned three weeks.

Additional experiments using a luciferase LNCaP (luc-LNCaP) cell line with the same cell inoculum was studied over the course of 8 weeks. Large tumors formed in two out of three of the mice after 8 weeks, reaching sizes of ~1 cm, while control mice did not develop tumors. This experiment demonstrates that the mice in the previous study very likely did not have sufficient time to develop tumors and would have if the experiment had been conducted over a longer time frame. In addition, these mice were imaged via bioluminescence for the presence of metastases (Supplementary Fig. 3). No metastases were present in either of the two mice that had tumors, indicating that the only source of PSA within the mouse serum is from the resected tumors.

Since the goal of this work was to monitor PSA levels in serum over time prior to tumor formation, preferably over multiple weeks, it was vital that the inoculation concentration of cancer cells was not too high. An inoculation of 1 × 10^6^ LNCaP cells, as previously described, can still be considered relatively high when factoring the overall volume of blood in an average mouse (approximately 2 mL). Thus, experiments were conducted to monitor tumor progression in mice using significantly reduced cell concentrations of 100,000 (100 k) and 10,000 (10 k) cells. To our knowledge, this is the first example of a mouse xenograft model using such a low concentration of LNCaP cells.

### Inoculation with 100 k Cells

**Figure 1:**
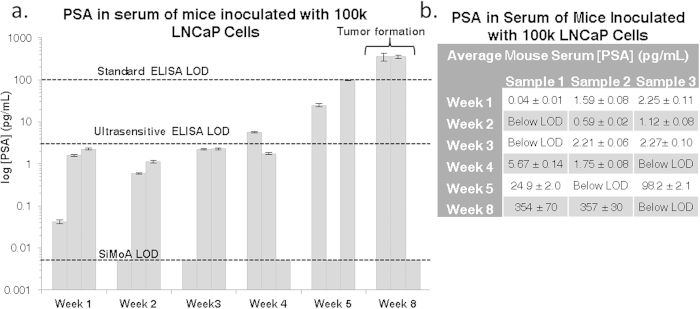
**a** Bar graph of measured PSA concentrations in the serum of mice inoculated with 100 k LNCaP cells on a log scale. All measurements were taken using serum from terminal bleeds of individual mice. Each sample was measured in triplicate. Error bars represent the standard deviation between triplicate measurements for each individual sample. An exponential increase in PSA concentration is observed over time. Three mice were examined at each time point, but several samples were below the 0.005 pg/mL detection limit for the assay. All samples with values below the LOD are plotted on the LOD line. LODs for standard ELISA and ultra-sensitive ELISA are shown for comparison at 100 pg/mL and 3 pg/mL. Values for each measurement are tabulated in **b**.

Eighteen NOD/SCID mice were subcutaneously injected with 100,000 LNCaP cells. Terminal bleeds were performed on three mice per week for a total of five weeks. The remaining three mice were sacrificed when palpable tumors formed (week 8). Replicates were used for each time point due to restrictions on the volume of whole blood that can be drawn from each mouse per week as well as the dead volume of the assay plates used for analysis, as described in the Materials and Methods section. Figure 1a illustrates the increase in PSA concentration in the serum of mice over time, where each time point represents a terminal bleed from an individual mouse. Supplementary Figure 4a demonstrates that the increase in PSA over time is exponential. Serum from three mice was measured for each time point and triplicate measurements were made for each sample.

Serum from six healthy male mice was tested and all samples had undetectable levels of PSA (not shown). This result is expected since mice do not express human PSA. Despite decreasing the number of cells used to inoculate the mice, the concentration of PSA in the serum of the majority of mice sacrificed after 1 to 5 weeks was measurable at values well above the SiMoA detection limit. All values measured with SiMoA prior to tumor formation were at or below the detection limit of commercially available ELISA kits. All measurements taken from weeks 1 to 3 were only measurable using SiMoA. Ultrasensitive ELISA was able to measure one sample in week 4 and both of the samples that were measurable by SiMoA from week 5. Two out of three mice developed large tumors (>8 mm in diameter) after eight weeks and had significantly elevated serum PSA levels (355 ± 2 pg/mL) compared to the rest of the mice. Notably, the mouse that did not develop a tumor after eight weeks also had undetectable levels of PSA. The mice with palpable tumors were the only samples with serum PSA concentrations that would have been easily detected using standard ELISA. PSA values for all mice are shown in Fig. 1b (mouse samples are arbitrarily labeled as 1, 2 and 3).

### Inoculation of 10 k Cells

**Figure 2:**
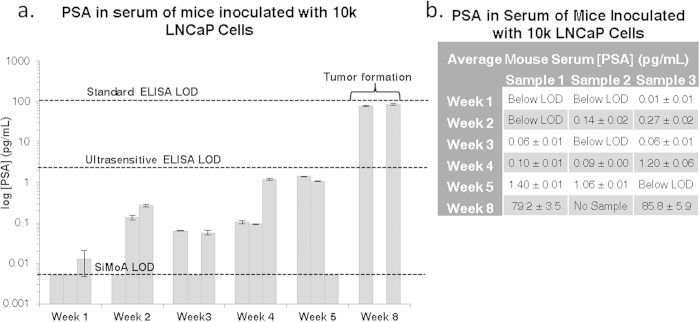
**a** Bar graph of measured PSA concentrations in the serum of mice inoculated with 10 k LNCaP cells on a log scale. All measurements were taken using serum from terminal bleeds of individual mice. Each sample was measured in triplicate. Error bars represent the standard deviation between triplicate measurements for each individual sample. An exponential increase in PSA concentration is observed over time. Three mice were examined at each time point, but several samples were below the 0.005 pg/mL detection limit of the assay. LODs for standard ELISA and ultra-sensitive ELISA are shown for comparison at 100 pg/mL and 3 pg/mL. Values for each measurement are tabulated in **b**.

In order to assess the sensitivity limits of SiMoA with our mouse model, the cell inoculate was further reduced to 10,000 LNCaP cells—*over 100 times lower than the typical dose used for inducing tumors.* A similar experimental approach was used, where 18 NOD/SCID mice were subcutaneously injected with 10,000 LNCaP cells and weekly terminal bleeds were performed. Small tumors (>3 mm in diameter) were present at week 8 in all three mice that were not sacrificed earlier; however, one mouse developed thymic lymphoma and it was not possible to obtain a serum sample (week 8, sample 2). Figure 2a depicts the increase in PSA concentration over time, with a large increase at week eight (average of 82.4 ± 4.7 pg/mL PSA), where palpable tumor formation occurred. As demonstrated in Supplementary Figure 4b, the increase in PSA over time is exponential. Several mice exhibited PSA concentrations below the SiMoA LOD, but measurements for at least two mice were recorded for each time point in triplicate. All measured PSA concentrations, including those with tumors, were below the detection limit of standard ELISA, as depicted on the graph, and only the samples containing tumors were detectable using ultra-sensitive ELISA.

### Tumor Characterization

**Figure 3:**
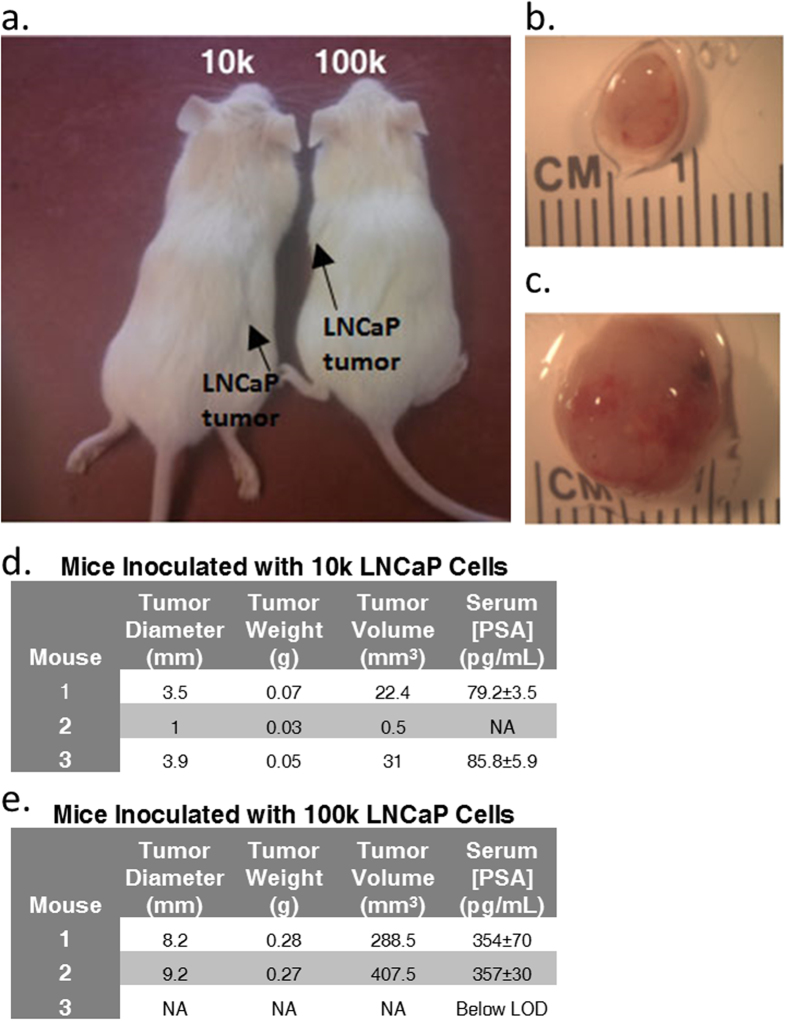
**a** Representative photographs of mice inoculated with 10 k and 100 k LNCaP cells. Photographs were taken after 8 weeks with tumor locations indicated by arrows. Photographs in **b-c** show representative tumors removed from mice inoculated with 10 k and 100 k LNCaP cells respectively, after 8 weeks at 8x magnification. Tables in **d-e** describe data pertaining to the tumors found in the 10 k and 100 k mouse cohorts, respectively, as well as the concentrations of PSA within the serum.

**Figure 4:**
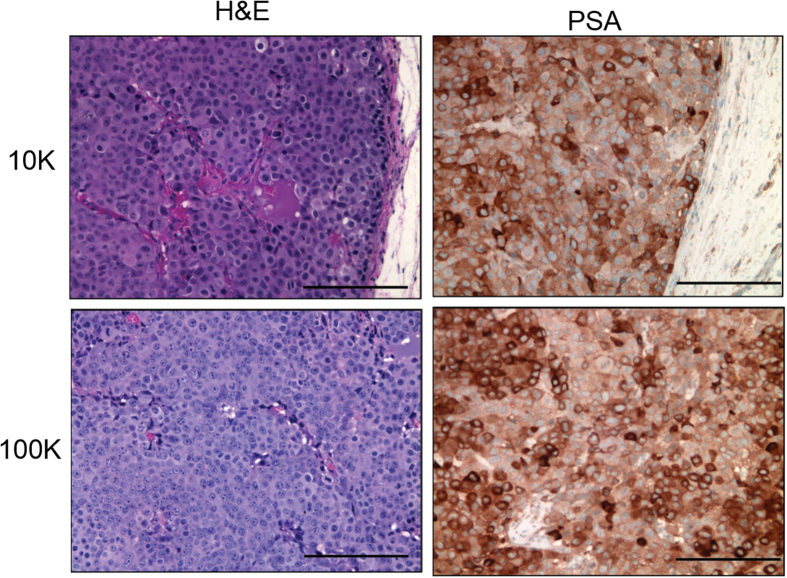
H&E and PSA staining of tumor samples from mice inoculated with both 10 k and 100 k LNCaP cells. PSA staining confirms the presence of PSA in the cells of the resected tumors. Magnification is 200x and scale bars are 100 μm.

As previously mentioned, mice from both the 10 k and 100 k cell cohorts developed tumors within eight weeks of inoculation. Images highlighting the location of tumors *in vivo* are shown in Fig. 3a. The tumors were removed for characterization and images of representative resected tumors are shown in Fig. 3b,c. Tables including tumor diameter, weight, volume, and serum PSA concentration are shown in Fig. 3d,e. The average PSA levels for each cohort were very similar, with an average of 82.4 ± 4.7 pg/mL and 355 ± 2 pg/mL for the 10 k and 100 k cohorts measured at week 8, respectively. Tumors were preserved in formalin and sections were stained with both hematoxylin and eosin (H&E) and PSA (Fig. 4). The PSA stain confirmed that the tumor cells were PSA positive.

## Discussion

SiMoA technology has the potential to revolutionize cancer diagnostics and therapeutics by non-invasively detecting cancer biomarkers in serum earlier than current methods. This work describes a proof-of-concept study where prostate cancer was induced in a mouse model at very low cell inoculums to demonstrate the utility of SiMoA as an early cancer detection tool. Increasing levels of PSA were measured in the serum of mice as a sign of tumor progression, since the only source of increasing levels of secreted human PSA in the mouse model is the proliferation of the PSA-secreting cancer cells. Several mice developed tumors, indicating that SiMoA can be used to monitor ultra-low levels of biomarkers in serum prior to the formation of palpable tumors.

In the case of mice inoculated with 100 k LNCaP cells, the PSA concentration after one week ranged from 0.04 to 2.25 pg/mL, levels much lower than the detection capabilities of conventional ELISA. In comparison, mice inoculated with only 10 k LNCaP cells resulted in even lower PSA concentrations, which were at or below the SiMoA LOD after week one. However, by week two, PSA measurements for the 10 k cohort surpassed the SiMoA LOD with obtained values of 0.20 ± 0.09 pg/mL, while those in the 100 k cohort yielded values of 1.71 ± 0.37 pg/mL after two weeks. The first samples with PSA concentrations high enough to be measured by conventional ELISA were collected eight weeks after inoculation; once palpable tumors formed. Although different mice were used for each measurement, the increasing trends of PSA concentration over the short time courses of these experiments within the majority of samples show that it is possible to monitor tumor markers during the initial stages of tumor formation in low volumes of serum using SiMoA. This sensitivity should enable the ability to detect the earliest stages of cancer, before palpable or visible tumors are detectable. Due to the limited number of cells used for inoculation, PSA was not detectable in all mouse replicates for either the 10 k or the 100 k model. Injecting mice with lower cell inoculums leads to issues regarding clonal heterogeneity, where the likelihood of having cancer stem cells or clones that can form tumors is reduced. The presence of low numbers of cancer stem cells is also the likely reason why only two of the 100 k mice developed tumors. This process has been described previously using LNCaP cells in the work of Wan and coworkers^20^. In addition, all mice are biologically unique and variations are expected between individuals.

The PSA concentrations that were detected in mice after tumor formation seemed to reasonably correlate with the number of cells used for inoculation in each mouse cohort. However, the PSA concentrations do not scale as may be expected (i.e. the concentration of PSA from mice with 100 k tumors was not 10x higher than the 10 k tumors). This result may be due to the fact that the tumors were encapsulated, thus hindering the secretion of PSA from the tumors into the bloodstream. Also, the presence of varying amounts of necrotic cells within the tumors could also result in different secretion rates of PSA. Images with samples of necrosis in tumors from each cohort are shown in Supplementary Figure 5. A larger sample size will be needed to further investigate this correlation.

The data presented here also demonstrate that, by utilizing SiMoA, significantly fewer LNCaP cells are required for PSA to be detected in serum. This methodology can be particularly useful when examining the growth of tumors in mice over time and at lower cell concentrations, where high cell concentrations were previously required. The technique could thus be utilized for models to study early tumor development, either for primary tumors or for relapse after treatment in more complex models. In addition, biomarker levels can potentially be monitored after chemotherapy to ascertain the efficacy of treatment. This work represents a proof-of-concept study using PSA; however, this work can easily be extended to use any other protein biomarker or cell line of interest, which would further advance the field of cancer diagnostics and early detection.

Through the creation of a low cell inoculum mouse model, we successfully demonstrated that SiMoA can be used to measure the biomarker PSA within the serum of mice that ultimately developed palpable tumors. The sensitive detection of circulating protein biomarkers can not only enable earlier detection of disease, but can also unmask the body’s unique underlying chemistry and offer more dynamic information that imaging techniques are incapable of providing. This work shows significant promise for the use of SiMoA in the field of oncology and early cancer detection as a non-invasive approach to early tumor detection. Future work involves creating panels of relevant biomarkers so that both tumor formation and the formation of biologically relevant disease can be predicted. The discrimination of biologically relevant tumors from those that would not become symptomatic could be incredibly powerful in reducing or eliminating the overtreatment of patients, which is a problem currently plaguing early detection methodologies^21^.

## Methods

### Mouse Xenografts

The human prostate cancer cell line LNCaP (ATCC CRL-1740) was provided by Dr. Charlotte Kuperwasser at Tufts Medical School. Cells were grown in RPMI 1640 media (Gibco) containing 10% fetal bovine serum and 1% antibiotic/antimycotic (Gibco). LNCaP cells were grown at 37 °C with 5% CO_2_ and were passaged for less than 3 weeks. The cells tested negative for mycoplasma (MilliPROBE; Millipore) and were authenticated by ATCC/Promega.

For injections, LNCaP cells were trypsinized (0.05%; Gibco) and counted using trypan blue to identify viable cells. Cells were resuspended in 50% v/v in Matrigel (BD Biosciences) and RPMI culture media for injections. Eight week old male NOD/SCID mice (Jackson Laboratories) were subcutaneously injected with 100 μL of resuspended cells. Mice were given food and water *ad libitum*. Whole blood was collected from mice under terminal anesthesia via cardiac puncture and transferred to SST Microtainer tubes (Becton, Dickinson, and Co.). The care of animals and all animal procedures were conducted in accordance with a protocol approved by the Tufts University Institutional Animal Care and Use Committee (IACUC).

Whole blood samples were allowed to clot on ice for 10 min. followed by centrifugation at 1,500 x g for 10 min at 4 °C. Serum was then removed and immediately snap frozen. All serum samples were stored at −80 °C prior to use. For SiMoA analysis, serum samples were diluted by a factor of four in PSA Diluent (Quanterix Corp.) before being loaded onto an automated HD-1 analyzer (Quanterix Corp.). Serum from healthy male mice was used as controls and was treated similarly. Tumor tissue was preserved in formalin and sections were stained with hematoxylin and eosin (H&E) and prostate specific antigen (PSA; Tufts Histology Core).

Due to restrictions imposed by the Tufts University IACUC, the maximum volume of whole blood that can be drawn from the NOD/SCID mice used is 7.5% of the animal’s total circulating blood, which is equivalent to ~82–105 μL per week. From this volume of whole blood, the maximum volume of serum obtained is at most 50 μL, which is equivalent to the dead volume of the plates used for these experiments. Due to these limitations, multiple mice were sacrificed per time point in order to obtain biological replicates and still obtain valuable information.

Lentiviral particles were generated by cotransfection of the pLenti-PGKV5LucNeo construct (Addgene) with pCMV-VSVG, expressing the vesicular stomatitis virus glycoprotein and the packaging construct pCMVΔR8.2Δvpr into 293T cells with FuGENE 6 transfection reagent (Promega). Lentivirus-containing supernatant from the transfected 293T cells was filtered through a 0.45 μm syringe filter and used to directly infect subconfluent LNCaP cells in the presence of 5 μg/mL protamine sulfate (Sigma). LNCaP-luc cells with lentiviral integration were selected with 750 μg/mL hygromycin.

To generate tumors, 1 × 10^6^ LNCaP-luc cells were injected subcutaneously in 100 μl of a 1:1 mixture of Matrigel and cell growth media into male 8 week old NOD/SCID mice. Control mice were injected with 100 μl of a 1:1 mixture of Matrigel and cell growth media only. When the tumors reached a diameter of 1 cm (after 8 weeks), all mice received a 100 μl intraperitoneal injection of 15 mg/mL luciferin. Five minutes after treatment, luminescence was quantified using an IVIS 200 Imager (Perkin Elmer). Bioluminescence was analyzed using Living Image software (Caliper Life Sciences).

### SiMoA PSA Assay

All single molecule measurements were taken using an HD-1 Analyzer (Quanterix Corp.). All HD-1 consumables, including wash buffers, sample diluent, assay discs, 96-well plates, sealing oil, cuvettes, and PSA reagents, were purchased from Quanterix Corp. The SiMoA platform has been described previously^9^,^22^. Briefly, a solution of approximately 5 × 10^6^ magnetic beads/mL in Tris buffer is loaded into the HD-1 analyzer. The beads are pre-coated with a mouse monoclonal PSA capture antibody. In addition to the beads, solutions of 0.3 μg/mL biotinylated detector antibody, 50 pM streptavidin β-galactosidase (sβg), and PSA sample diluent are also added to the HD-1 analyzer. The beads are added into a disposable cuvette via the instrument such that 100 μL of bead solution is loaded per sample. The beads are collected via magnetic separation, the supernatant is removed and 100 μL of either a protein standard or serum sample is added to the beads. The protein standard/sample is incubated with the beads for a total of 15 minutes. The beads are then washed 4 times with wash buffer and 100 μL of biotinylated detector antibody is added. The beads are incubated for a total of 5 minutes with the detector, followed by 4 washes. 100 μL of sβg is then added to the beads and incubated for 5 minutes, followed by eight washes. Finally, the enzyme substrate (resorufin β-D galactopyranoside) is added to the beads and the mixture is loaded onto a disc containing an array of 216,000 microwells and sealed with oil. Both fluorescence and white light images are taken of each well. Wells that contain a bead and increase in fluorescence intensity are considered a positive signal. The average number of enzymes per bead (AEB) is calculated from the fraction of active wells, which is correlated to the concentration of analyte^10^. The assay LOD was calculated by extrapolating the background PSA concentration plus 3 standard deviations of the background using a 4-parameter logistic fit.

## Additional Information

**How to cite this article**: Schubert, S. M. *et al.* Ultra-sensitive protein detection via Single Molecule Arrays towards early stage cancer monitoring. *Sci. Rep.* **5**, 11034; doi: 10.1038/srep11034 (2015).

## Supplementary Material

Supplementary Information

## References

[b1] Etzioni R. *et al.* Quantifying the role of PSA screening in the US prostate cancer mortality decline. Cancer Causes Control 19, 175–181 (2008). 18027095 10.1007/s10552-007-9083-8 PMC3064270

[b2] Berry D. A. *et al.* Effect of Screening and Adjuvant Therapy on Mortality from Breast Cancer. N. Engl. J. Med. 353, 1784–1792 (2005). 16251534 10.1056/NEJMoa050518

[b3] Shapira I. *et al.* Circulating biomarkers for detection of ovarian cancer and predicting cancer outcomes. Br J Cancer 110, 976–983 (2014). 24366298 10.1038/bjc.2013.795 PMC3929876

[b4] Edwards B. K. *et al.* Annual report to the nation on the status of cancer, 1975-2006, featuring colorectal cancer trends and impact of interventions (risk factors, screening, and treatment) to reduce future rates. Cancer 116, 544–573 (2010). 19998273 10.1002/cncr.24760 PMC3619726

[b5] Paoletti C. & Hayes D. F. Molecular Testing in Breast Cancer. Annu. Rev. Med. 65, 95–110 (2014). 24422569 10.1146/annurev-med-070912-143853

[b6] Nolen B. M. *et al.* Prediagnostic Serum Biomarkers as Early Detection Tools for Pancreatic Cancer in a Large Prospective Cohort Study. PLoS ONE 9, e94928 (2014). 24747429 10.1371/journal.pone.0094928 PMC3991628

[b7] Paik S. *et al.* A Multigene Assay to Predict Recurrence of Tamoxifen-Treated, Node-Negative Breast Cancer. N. Engl. J. Med. 351, 2817–2826 (2004). 15591335 10.1056/NEJMoa041588

[b8] Anderson N. L. & Anderson N. G. The Human Plasma Proteome: History, Character, and Diagnostic Prospects. Molecular & Cellular Proteomics 1, 845–867 (2002). 12488461 10.1074/mcp.r200007-mcp200

[b9] Rissin D. M. *et al.* Single-molecule enzyme-linked immunosorbent assay detects serum proteins at subfemtomolar concentrations. Nat. Biotechnol. 28, 595–599 (2010). 20495550 10.1038/nbt.1641 PMC2919230

[b10] Chang L. *et al.* Single molecule enzyme-linked immunosorbent assays: Theoretical considerations. J. Immunol. Methods 378, 102–115 (2012). 22370429 10.1016/j.jim.2012.02.011 PMC3327511

[b11] Ferguson R. A., Yu H., Kalyvas M., Zammit S. & Diamandis E. P. Ultrasensitive detection of prostate-specific antigen by a time-resolved immunofluorometric assay and the Immulite immunochemiluminescent third-generation assay: potential applications in prostate and breast cancers. Clin. Chem. 42, 675–684 (1996). 8653891

[b12] Liu D. *et al.* Glucose Oxidase-Catalyzed Growth of Gold Nanoparticles Enables Quantitative Detection of Attomolar Cancer Biomarkers. Anal. Chem. 86, 5800–5806 (2014). 24896231 10.1021/ac500478g PMC4066917

[b13] Malhotra R. *et al.* Ultrasensitive Detection of Cancer Biomarkers in the Clinic by Use of a Nanostructured Microfluidic Array. Anal. Chem. 84, 6249–6255 (2012). 22697359 10.1021/ac301392g PMC3418660

[b14] Li F. *et al.* An ultrasensitive sandwich-type electrochemical immunosensor based on signal amplification strategy of gold nanoparticles functionalized magnetic multi-walled carbon nanotubes loaded with lead ions. Biosensors and Bioelectronics 68, 626–632 (2015). 25656779 10.1016/j.bios.2015.01.049

[b15] Zhang J. *et al.* Novel Signal-Enhancing Immunoassay for Ultrasensitive Biomarker Detection Based on Laser-Induced Fluorescence. Anal. Chem. 87, 2959–2965 (2015). 25655002 10.1021/ac504515g

[b16] Wilson D. H. *et al.* Fifth-Generation Digital Immunoassay for Prostate-Specific Antigen by Single Molecule Array Technology. Clin. Chem. 57, 1712–1721 (2011). 21998342 10.1373/clinchem.2011.169540 PMC3402036

[b17] Song L. *et al.* Single molecule measurements of tumor necrosis factor α and interleukin-6 in the plasma of patients with Crohn’s disease. J. Immunol. Methods 372, 177–186 (2011). 21821036 10.1016/j.jim.2011.07.015

[b18] Warren A. D. *et al.* Disease Detection by Ultrasensitive Quantification of Microdosed Synthetic Urinary Biomarkers. J. Am. Chem. Soc. 136, 13709–13714 (2014). 25198059 10.1021/ja505676h PMC4183649

[b19] Horoszewicz J. S. *et al.* LNCaP Model of Human Prostatic Carcinoma. Cancer Res. 43, 1809–1818 (1983). 6831420

[b20] Wan X. S., Zhou Z., Steele V., Kopelovich L. & Kennedy A. R. Establishment and characterization of sublines of LNCaP human prostate cancer cells. Oncology Reports 10, 1569–1575 (2003). 12883743

[b21] Esserman L. J. *et al.* Addressing overdiagnosis and overtreatment in cancer: a prescription for change. Lancet Oncol 15, e234–e242 (2014). 24807866 10.1016/S1470-2045(13)70598-9 PMC4322920

[b22] Kan C. W. *et al.* Isolation and detection of single molecules on paramagnetic beads using sequential fluid flows in microfabricated polymer array assemblies. Lab on a Chip 12, 977–985 (2012). 22179487 10.1039/c2lc20744c

