# Supplementary material for: Ultra-sensitive protein detection via Single Molecule Arrays towards early stage cancer monitoring
Source: Sci Rep. 2015 Jun 8;5:11034. doi: 10.1038/srep11034 (PMC4458912; doi:10.1038/srep11034)
Supplement: Supplementary Information [file srep11034-s1.pdf]

Supplementary Information to Ultra-sensitive protein detection via Single Molecule Arrays towards early stage cancer monitoring

Stephanie M. Schubert, Lisa M. Arendt, Wenhui Zhou, Shazia Baig, Stephanie R. Walter, Rachel J. Buchsbaum, Charlotte Kuperwasser, and David R. Walt*

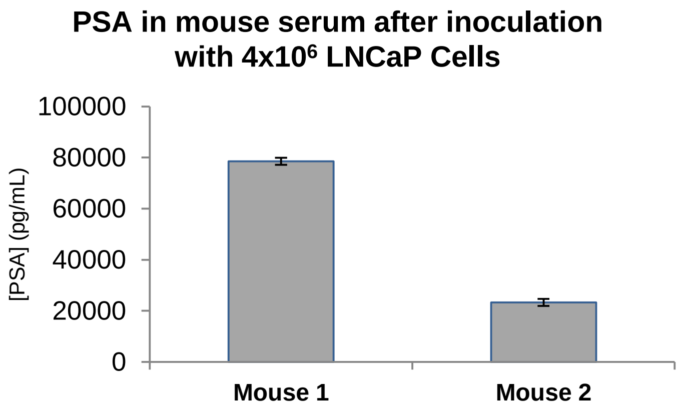

Supplementary Figure 1. Bar graph of measured PSA concentrations in the serum of mice inoculated with $4x10^6$ LNCaP cells. Measurements were taken four weeks after inoculation. Both measurements were well above the detection limits of standard ELISA and beyond the scope of SiMoA. Each sample was measured in triplicate. Error bars represent the standard deviation between triplicate measurements for each individual sample

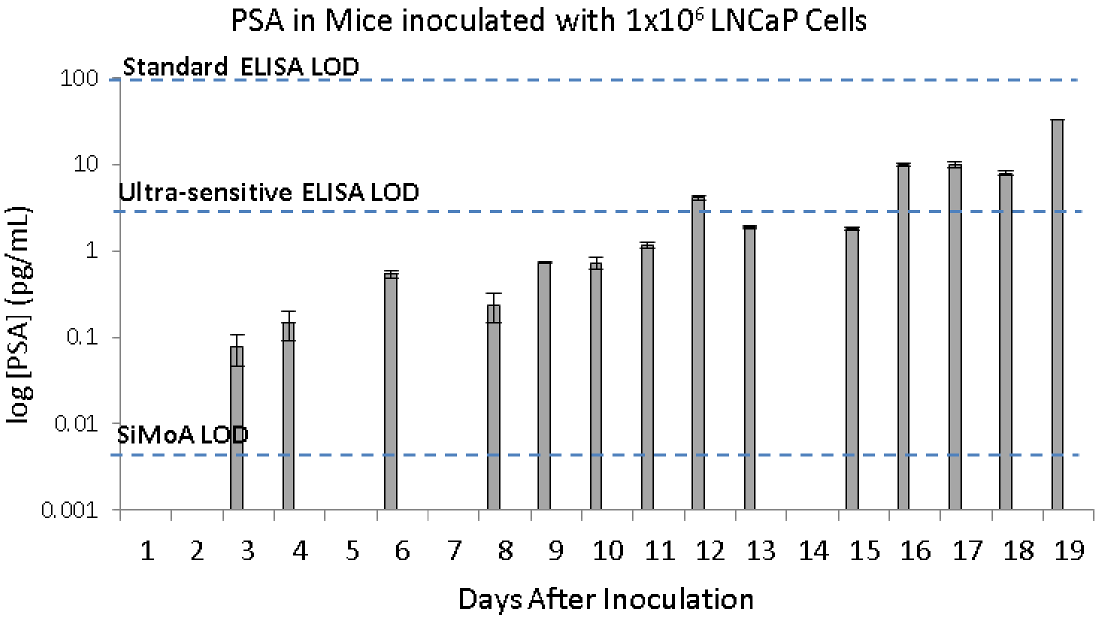

Supplementary Figure 2. Bar graph showing the log of PSA concentrations in the serum of mice inoculated with $1x10^6$ LNCaP cells over 19 days. All measurements were taken using serum from terminal bleeds of individual mice where one mouse was sacrificed for each time point. A general increase in PSA concentration is displayed over time from days 1-19. The assay SiMoA LOD was 0.005 pg/mL. For comparison, the LOD of ultrasensitive (3 pg/mL) and standard ELISA (100 pg/mL) are shown. Each sample was measured in triplicate. Error bars represent the standard deviation between triplicate measurements for each individual sample.

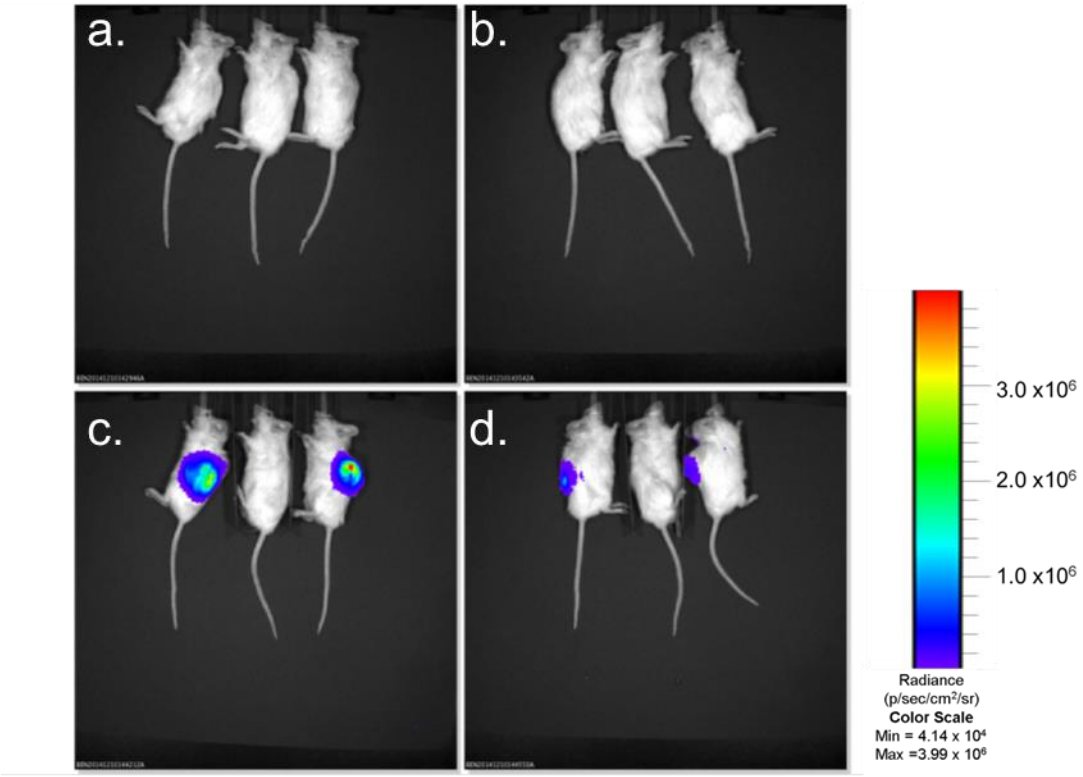

Supplementary Figure 3. Bioluminescence images of three NOD/SCID mice inoculated with $1x10^6$ luc-LNCaP cells and three control mice injected with Matrigel and cell growth media after 8 weeks. The control mice showed no signs of tumor formation in either the left (**a**) or right (**b**) views; however, the mice injected with the luc-LNCaP cells had 1 cm tumors that were clearly visible from both the left (**c**) and right (**d**) views of the animal. No evidence of metastasis is present among any of the three mice. The middle mouse did not grow a tumor.

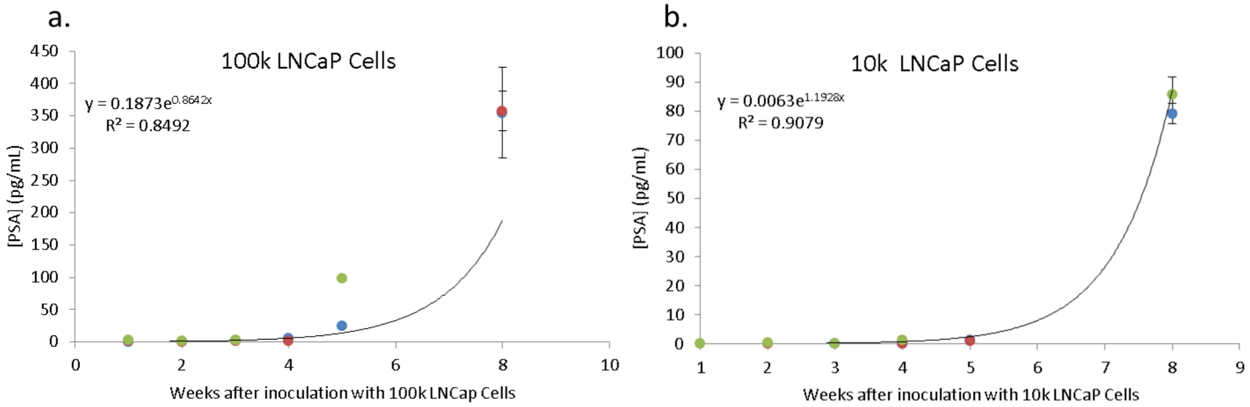

Supplementary Figure 4. Scatter plots showing the exponential increase of PSA over time. Mice were inoculated with a) 100k and b) 10k LNCaP cells. Each sample was measured in triplicate. Error bars represent the standard deviation between triplicate measurements for each individual sample.

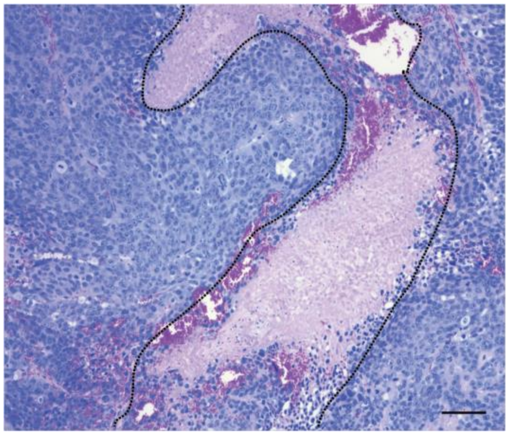

Supplementary Figure 5. Representative photo of H&E stained tumor from mice inoculated with 100k LNCaP cells at 100x magnification. The necrotic area is highlighted via the dotted line. Scale bar is 100 µm. The presence and extent of necrosis in the tumors from each mouse may lead to varying PSA concentrations in the respective serum.
